# Daily intake of cuminaldehyde-rich cumin essential oil improves cognitive function in healthy elderly Japanese adults: a randomized, double-blind, placebo-controlled pilot study

**DOI:** 10.3389/fnut.2026.1784027

**Published:** 2026-04-20

**Authors:** Kengo Ito, Jieun Yoon, Kazunori Sasaki, Yuri Nakagawa, Hiroyuki Onda, Hiroko Isoda, Tomohiro Okura

**Affiliations:** 1 S&B FOODS Inc., Itabashi City, Tokyo, Japan; 2 Institute of Health and Sport Sciences, University of Tsukuba, Tsukuba, Japan; 3 R&D Center for Tailor-Made QOL, University of Tsukuba, Tsukuba, Japan; 4 The Alliance for Research on the Mediterranean and North Africa (ARENA), University of Tsukuba, Tsukuba, Japan; 5 Open Innovation Laboratory for Food and Medicinal Resource Engineering, National Institute of Advanced Industrial Science and Technology (AIST), Tsukuba, Japan; 6 Institute of Life and Environmental Sciences, University of Tsukuba, Tsukuba, Japan

**Keywords:** anti-inflammatory, cognitive function, cumin, cuminaldehyde, functional food, randomized clinical trial

## Abstract

**Background:**

To extend healthspan has risen demand to maintain cognitive function through the exploitation of bioactive compounds from natural sources. We assessed the effects of daily intake of cumin essential oil (CEO), primarily composed of cuminaldehyde, on cognitive function in the elderly.

**Methods:**

Thirty-eight participants (ages 65–86) were randomly assigned to either the CEO group (*n* = 19) or the placebo group (*n* = 19) for a 12-week trial (UMIN no. 000050328). Cognitive performance on Cognitrax test and relevant blood biomarkers were assessed before and after the intervention. Repeated measures analysis of covariance, adjusted for age and years of education, was performed.

**Results:**

The analysis revealed a significant group × time interaction for the CEO group in psychomotor speed (*p* = 0.012) and reaction time (*p* = 0.022). Additionally, a simple main effect analysis indicated that psychomotor speed and reaction time scores were significantly improved in the CEO group post-intervention compared to pre-intervention (*p* = 0.048 and *p* = 0.012, respectively). Exploratory biomarker analyses showed significant main effects of time; however, no significant group × time interactions were observed for any biomarker, suggesting that these biological changes were not uniquely attributable to CEO supplementation in this pilot study.

**Conclusion:**

Overall, this study suggest that the daily intake of CEO, rich in cuminaldehyde, have a beneficial effect on cognitive function in the elderly. These findings provide foundational knowledge that could contribute to strategies aimed at extending the healthy life expectancy of older adults.

**Clinical trial registration:**

https://center6.umin.ac.jp/cgi-open-bin/ctr/ctr_view.cgi?recptno=R000057216, identifier UMIN000050328.

## 1 Introduction

Life expectancy at birth for Japanese men and women in 2050 is projected to be 84.0 and 90.4 years, respectively (1). As efforts to extend lifespan continue, age-related diseases are receiving increasing attention in the context of maintaining health during aging. Advancing age is the greatest risk factor for chronic degenerative diseases (2, 3). Even in healthy older adults, cognitive decline and neurodegenerative changes are commonly observed (4). According to Livingston et al. (5, 6) there are 12 modifiable risk factors that could potentially prevent or delay up to 40% of dementia cases, although factors such as diet and sleep, which may also be significant, were not fully addressed in their model.

Recently, there has been growing research interest in specific lifestyles, diets, and certain ingredients, particularly those associated with the Mediterranean region, due to their potential effects on cognitive function (7). *Cuminum cyminum* (cumin), a widely used culinary spice belonging to the Apiaceae family and native to the Mediterranean region, is primarily utilized in the form of seeds, which are botanically classified as fruits (8). Cumin seeds are popular in various countries for their distinctive aroma and are commonly used as a food ingredient and seasoning in dishes such as curry.

Cumin seeds have also been utilized in traditional medicine for centuries, particularly in countries like India. Considering the major health challenges in modern society, including diabetes, arthritis, cardiovascular diseases, and cancer, the anti-proliferative, anti-diabetic, anti-inflammatory, and anti-hypercholesterolemic effects of spices have underscored their significant role in traditional medicine (9). Experimental studies have demonstrated that cumin exhibits anti-inflammatory (10), anti-oxidant and memory-enhancing effect (11). Specifically, cuminaldehyde, a key component of CEO, has been reported as a potential therapeutic agent against cytotoxic processes involved in neurodegenerative diseases (12). TNF-*α* and IL-6 are proinflammatory cytokines expressed in microglia, astrocytes, and neurons in brain tissue and mononuclear cells in peripheral blood. Elevated levels of these cytokines have been linked to neurodegenerative diseases, such as Alzheimer’s disease and age-related cognitive decline (13, 14). The plasma Aβ_1-40_/Aβ_1-42_ratio serves as a critical biomarker for Alzheimer’s disease and dementia, with an elevated ratio being a recognized risk factor for disease progression (15, 16). BDNF, a neurotrophin, plays a crucial role in neuronal survival, proliferation, differentiation, and synaptic plasticity in the brain (17). Reduced plasma BDNF levels have been associated with depression and have also been proposed as a potential biomarker for Alzheimer’s disease and dementia (18, 19).

Furthermore, a study by Ng et al. (20) reported that individuals who consumed curry, a dish typically containing cumin, more than once every 6 months had better Mini-Mental State Examination (MMSE) scores compared to those who consumed it never or less frequently. However, the complexity of spices in curry makes it challenging to establish a strong causal relationship between their consumption and cognitive benefits. Therefore, a crucial research question of this study was whether the consumption of CEO, which is rich in cuminaldehyde, has a beneficial effect on the cognitive function of older adults.

## 2 Materials and methods

### 2.1 Ethical statement and study organization

The study was conducted in accordance with ethical guidelines outlined in the Declaration of Helsinki and its subsequent amendments (21). All eligible participants were thoroughly informed about the study’s objectives, design, inclusion and exclusion criteria, supplement intervention, assessment procedures, insurance coverage for potential injuries, withdrawal rights, and privacy protection measures. All participants provided written informed consent.

This study was approved by the ethics committee of the Institutional review board of Chiyoda Paramedical Care Clinic (October 21, 2022; reference no. 22102104). Participant recruitment and data management were entrusted to a contracted research agency (e-Sport Corporation, Tsukuba, Japan). The study protocol was registered with the University Hospital Medical Information Network Center (UMIN no. 000050328). All measurements were performed at the Innovation Medical Research Institute in University of Tsukuba.

### 2.2 Study design and criteria

This randomized, double-blind, placebo-controlled, parallel-group study was conducted over a 12-week period from February 13, 2023, to May 7, 2023. Participants underwent assessments at baseline (week 0) and at the end of the study (week 13). The target sample size was determined based on the sample size calculation of a previous study (22), which recommended a minimum of 50 for a pilot study.

Elderly residents of Tsukuba City, Japan, were recruited over 1 month through leaflet distribution and snowball sampling. A screening survey was conducted via telephone interviews using self-reported general health questionnaires. The inclusion criteria were as follows:

(1) Individuals aged 65–89 at the time of obtaining informed consent.
(2) Those with no history of diabetes, cerebrovascular disease, cardiovascular disease, cancer, or paralysis.
(3) Those who can intake cumin essential oil (no allergies or intolerance).
(4) Those who have no allergy, intolerance, or food restriction to gelatin.
(5) Those who consume curry powder-based foods but can adjust their intake to no more than 2–3 times per week during the study (including curry with rice, curry udon, and curry bread).
(6) Those who have not participated or are not currently participating in any other clinical research within the past 3 months.
(7) Those who fully understand the content of this study and can commute to the study site on their own.

The exclusion criteria were as follows:

(1) Individuals diagnosed with dementia (MMSE ≤ 23 points) or depression (Geriatric Depression Scale ≥ 11 points).
(2) Individuals currently taking medications for dementia or depression.

Participants were instructed to keep a diary to record their adherence to the intake of capsules and consumption of curry powder-based foods.

**Figure 1:**
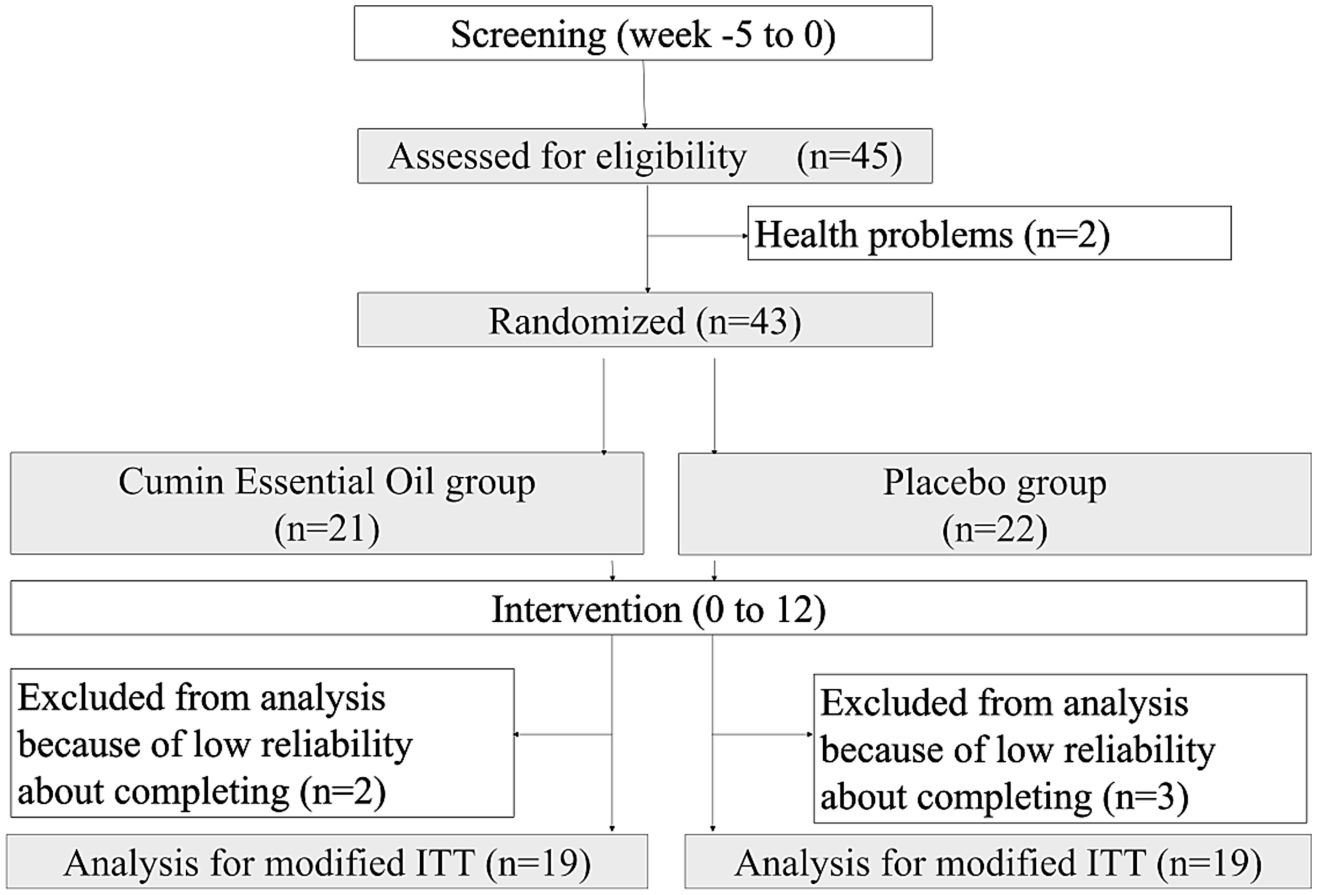
Randomized controlled trial flowchart of this study (modified ITT).

Randomization was performed by an independent third party who was not involved in recruitment, assessment, intervention delivery, or data analysis. We used stratified randomization by sex and age to ensure balance between groups. Allocation was concealed using sequentially numbered, opaque, sealed envelopes (or an equivalent procedure), and the randomization list was not accessible to the investigators until the database was locked. Participants, investigators, outcome assessors, and data analysts remained blinded to group assignments throughout the trial. The study flowchart is shown in Figure 1.

### 2.3 Cumin essential oil and placebo samples

Cumin essential oil (Nihonkaken Co., Ltd., Tokyo) was obtained from Indian cumin seeds through steam distillation, resulting in an oil comprising uminaldehyde. The test food used in this study was a cumin capsule formulated with CEO, containing 25 mg of cuminaldehyde per capsule. A placebo capsule was prepared to closely match the cumin capsule in appearance, taste, and other physical properties.

Participants in the CEO group were instructed to take one cumin capsule daily after breakfast with water, while those in the placebo group consumed the placebo under the same conditions.

The composition of the test formulations was as follows: the CEO group received capsules containing 15.25% cumin essential oil and 34.75% medium-chain triglyceride oil, whereas the placebo group received capsules containing 50% medium-chain triglyceride oil as a substitute (Table 1).

**Table 1:** Cumin essential oil capsule and placebo capsule.

Group	Form	Ingredients	Contents ratio (%)
CEO	Soft capsule	Cumin essential oil	15.3
		Medium-chain-triglyceride oil	34.8
		Gelatin	33.8
		Food additive grade glycerin	13.5
		Caramel pigment	2.7
		Total	100
Placebo	Soft capsule	Medium-chain-triglyceride oil	50.0
		Gelatin	33.8
		Food additive grade glycerin	13.5
		Caramel pigment	2.7
		Total	100

### 2.4 Component analysis

Gas chromatography–mass spectrometry analysis was performed using a GCMS-TQ8040 system (SHIMADZU CORPORATION, Japan). A Stabilwax column (0.25 mm I. D., 60 m length, 0.25 μm film thickness; Restek, United States) was employed with the following temperature program: an initial temperature of 60 °C was maintained for 4 min, followed by an increase to 100 °C at a rate of 10 °C/min with a 1-min hold. The temperature was then further increased to 200 °C at 5 °C/min, followed by a final ramp to 230 °C at 7 °C/min, where it was held for 10 min. Helium was used as the carrier gas at a constant flow rate of 1.0 mL/min. The interface temperature was set at 230 °C, while the ion source temperature was maintained at 200 °*C. electron* ionization was performed at 70 eV. The selected ions for each compound are listed in Table 2. For sample preparation, 10 mg of CEO was dissolved in hexane to a final volume of 10 mL. A 1 μL aliquot of the prepared sample was injected into a GC-2010 Plus system (SHIMADZU CORPORATION, Japan).

**Table 2:** Concentration of components of CEO.

Components	*m/z*	%(w/w)
β-pinene	93*, 91**, 63**	11.3
γ-terpinene	93*, 91**, 136**	10.5
p-cymen	119*, 91**, 134**	11.0
cuminaldehyde	133*, 148**, 105**	43.0
α-terpiene	121*, 93**, 136**	<0.05
terpinolene	93*, 121**, 136**	0.05

Limits of quantitation were 0.05%, respectively. *Quantitative ion, **Qualitative ions.

### 2.5 Primary outcome

The Cognitrax test (Health Solution, Inc., Japan) was used to assess the cognitive function of the participants after the intervention. The Cognitrax scores are standardized based on data from a large population of individuals aged 7–90 years (23). This computerized test battery is designed to assesses following multiple cognitive domains and subsequently provides quantitative scores:

- Composite memory: Measures the ability to recognize, retain, and retrieve both verbal and visual information, including words and geometric figures.
- Verbal memory: Evaluates recognition, retention, and retrieval of words.
- Visual memory: Assesses recognition, retention, and retrieval of geometric figures.
- Psychomotor speed: Examines the ability to perceive, attend to, and respond to visual-perceptual information, as well as perform motor speed and fine motor coordination tasks.
- Reaction time: Determines how quickly an individual responds (in milliseconds) to simple and progressively complex instructions.
- Complex attention: Measures the ability to track and respond to multiple stimuli over extended periods, as well as the capacity for rapid and accurate mental task execution requiring vigilance.
- Cognitive flexibility: Assesses adaptability to rapidly changing and increasingly complex instructions, as well as the ability to manipulate information effectively.
- Processing speed: Evaluates the efficiency of information recognition and processing, including perception, attention, response execution, motor speed, fine motor coordination, and visual-perceptual abilities.
- Executive function: Measures how well an individual recognizes rules, categories, and manages or navigates rapid decision making.
- Simple attention: Measures the ability to track and respond to a single defined stimulus over lengthy periods of time while performing vigilance and response inhibition tasks quickly and accurately.
- Motor speed: Assesses the ability to perform movements to produce and satisfy an intention toward a manual action and goal.

### 2.6 Secondary outcomes

Blood samples were collected between 7:00 and 9:00 a.m. after a minimum fasting period of 12 h. Venipuncture was performed at the median cubital vein, and blood was drawn into EDTA 2 K (5 mL) and EDTA 2Na (7 mL) vacuum blood collection tubes. The samples were left at room temperature for 30 min until complete coagulation, followed by centrifugation at 3,000 rpm for 10 min. Hematological and biochemical parameters, including diacron reactive oxygen metabolites (d-ROMs) and the biological antioxidant potential (BAP), were analyzed at Kotobiken Medical Laboratories, Inc., Japan. Plasma concentrations of proinflammatory cytokines tumor necrosis factor-*α* (TNF-α) and interleukin-6 (IL-6), amyloid *β* (Aβ)_1–40_ and Aβ_1-42_, and brain-derived neurotrophic factor (BDNF) were quantified using commercial enzyme-linked immunosorbent assay kits (TNF-α, IL-6, and BDNF: Proteintech group Inc., Japan; Aβ_1-40_ and Aβ_1-42_: FUJIFILM Wako Pure Chemical Corporation, Japan). The enzyme-linked immunosorbent assay analysis was performed according to the manufacturer’s instructions, with all plasma samples diluted 1:2 using the provided diluent. Each sample and standard were analyzed in duplicate to ensure accuracy.

Trail Making Peg tests were carried out (24) as the test for handily evaluating cognitive function.

Quality of life were evaluated by geriatric depression scale, Physical Activity Scale for Elderly, Profile of Mood States 2nd Edition short version: 18 years or above, Pittsburgh Sleep Quality Index and MOS 8-item short form Healthy survey followed the manual (25).

### 2.7 Evaluation of the neuroprotective effect of CEO

The human neuroblastoma SH-SY5Y cell line was purchased from the American Type Culture Collection. SH-SY5Y cells were cultured in a 1:1 (v/v) mixture of Dulbecco’s modified Eagle Medium and Ham’s F-12 medium (FUJIFILM Wako Pure Chemical Corporation, Japan) supplemented with 15% heat-inactivated fetal bovine serum (Bio West, United States) and 1% penicillin (5,000 μg/mL)-streptomycin (5,000 IU/mL) (PS) (FUJIFILM Wako Pure Chemical Corporation, Japan) at 37 °C in a humidified atmosphere of 5% CO_2_ in air. SH-SY5Y cells were cultured in 100-mm petri dishes or 96-well plates. Serum-free Eagle’s minimum essential medium (Gibco, Japan). Cell viability and mitochondrial activity were determined using a 3-(4,5-dimethylthiazol-2-yl)-2,5-diphenyltetrazolium bromide (MTT) assay to check for effects of CEO, cuminaldehyde and Aβ42 on cytotoxicity. SH-SY5Y cells were seeded at 2 × 10^5^ cells/mL in 96-well plates (BD BioCoat, United States) and incubated for 24 h. To evaluate the neuroprotective effects of CEO and cuminaldehyde against Aβ-induced cytotoxicity, SH-SY5Y cells were pre-treated with CEO (25 and 50 μg/mL) or cuminaldehyde (3.705 and 7.41 μg/mL) for 24 h before 2.5 μM Aβ_42_ monomer treatment. After 24 h Aβ_42_ treatment, a solution of 5 mg/mL MTT dissolved in PBS was added (10 μL/well) and incubated for another 24 h. The resulting MTT formazan was dissolved in 100 μL of 10% SDS (FUJIFILM Wako Pure Chemical Corporation, Japan) and the absorbance was measured using a multi-detection microplate reader (Varioskan LUX VL0000D0, Thermo Fisher Scientific K. K., Japan). Results of *in vitro* study are expressed as the mean ± standard deviation. Statistical analysis of the control and treatment groups was conducted using one-way ANOVA with the GraphPad Prism 9 software (Dotmatics, San Diego, CA, United States). Statistical significance was set at *p* < 0.05. The results represent the mean of three separate experiments.

### 2.8 Statistical analysis

Initially, data cleaning was conducted for cognitive function measures as the primary outcome. Then, the study population was defined. Invalid values were handled as missing values. The invalid values were identified through the observation of measurement outliers greater than two standard deviations (SD) from the mean in both directions and exhibited substantial changes. The post-point missing value was imputed as the pre-point measurement value. Finally, the participants who had missing data in the cognitive function measurement were excluded from the analysis due to low reliability of their completion of the Cognitrax assessment. For the post-hoc analysis on biomarkers as a secondary outcome, the same handling data was additionally conducted on each outcome.

All statistical analyses were performed using SPSS (version 26, IBM, Armonk, NY, United States), with a significance set at *p* < 0.05. Baseline characteristics were summarized as mean ± SD for continuous variables and as frequency counts for categorical variables. Comparisons between groups were conducted using Student’s *t*-test for normally distributed continuous variables and the χ^2^ test for categorical variables.

To assess differences in all outcome measures over time, repeated measures analysis of covariance was performed, adjusting for age and years of education to evaluate within- and between-group differences, considering two levels for the time factor (pre- and post-trial) and two levels for the group factor (CEO group and placebo group). After performing tests for normality, variables for which normality could not be rejected were additionally analyzed with Mann-Whitney_U test to examine the robustness of the results as between-group comparisons (Supplementary Tables 3, 4). In the event that a significant difference was observed between the groups with respect participant’s characteristics, the other potential covariates were considered. When a significant interaction between time and group was detected, *post hoc* analyses were conducted to assess the effect of time within each group. If no significant interaction was observed, the main effects of time and group were analyzed independently. To quantify the magnitude of changes between pre- and post-trial assessments, effect sizes (Cohen’s *d*) were calculated based on the mean change as follows formula:


$$\text{Cohe}n^{’}s\;d=(\mathrm{mea}n_{\text{post}}-\mathrm{mea}n_{\mathrm{pre}})÷\surd ((SD_{\text{post}}^{2}+SD_{\mathrm{pre}}^{2})÷2)$$

## 3 Results

### 3.1 Concentration of components

**Figure 2:**
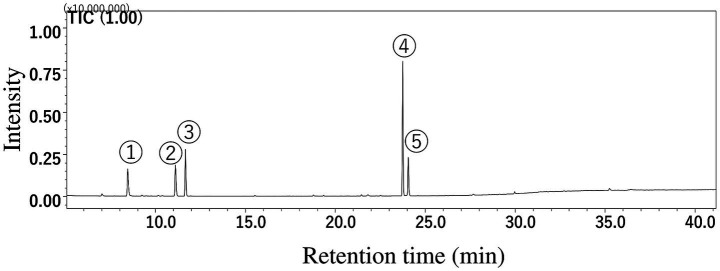
The cumin essential oil was analyzed using GC/MS. The chromatogram show following 5 major peaks: ① β-pinene, ② *γ*-terpinene, ③ *p*-cymene, ④ cuminaldehyde, ⑤ 1,3-*p*-menthadien-7-al.

Gas chromatography–mass spectrometry analysis determined the composition of the CEO used in the clinical trial. Gas chromatography–mass spectrometry analysis revealed five dominant components in CEO (Figure 2), but 1,3-*p-*Menthadien-7-al was not quantified due to the unavailability of a reference standard. In addition to these components, *α*-terpinene and terpinolene were reported to exert protective effects on neuroblastoma cell (26). Among the identified components, cuminaldehyde (43.0%) was the most abundant, followed by *β*-pinene (11.3%), *p*-cymene (11.0%), *γ*-terpinene (10.5%), and terpinolene (0.05%). The detailed composition is presented in Table 2 and Figure 2.

### 3.2 Participants characteristics

Baseline participant characteristics are presented in Table 3. A total of 43 participants were randomly assigned to either the CEO group (*n* = 21) or placebo group (*n* = 22) for a 12-week trial. However, five participants from both groups were excluded from the final analysis due to low reliability in completing the Cognitrax assessment (Figure 1). The overall compliance rate was 96.8%, with an intake rate of 94.8% in the CEO group and 98.7% in the placebo group. No significant differences in demographic or clinical variables were observed between the two groups at baseline (Table 3). The mean age of participants was 74.7 years (range: 65–86 years).

**Table 3:** Characteristics of participants by each group.

Variables	All (*n* = 38)		CEO group (*n* = 19)		Placebo group (*n* = 19)		*P*-value
	Range	Mean ± SD	Range	Mean ± SD	Range	Mean ± SD	
Age (years)^†^	65–86	74.7 ± 5.4	67–83	75.3 ± 5.0	65–86	74.1 ± 5.8	0.513^†^
Height (cm)^†^	142.6–172.5	158.5 ± 8.3	145.8–171.7	158.5 ± 8.5	142.6–172.5	158.5 ± 8.3	0.997^†^
Body weight (kg)^†^	36.9–78.8	57.5 ± 9.9	40.6–71.8	56.9 ± 9.2	36.9–78.8	58.1 ± 10.7	0.714^†^
Body mass index (kg/m^2^)^†^	14.8–26.5	22.7 ± 2.6	18.1–25.9	22.5 ± 2.3	14.8–26.5	22.7 ± 2.8	0.597^†^
MMSE (points)	24–30	28.7 ± 1.5	24–30	28.7 ± 1.6	26–30	28.8 ± 1.3	0.588†
Education [n (%)]^a^							0.503^a^
Middle school	1 (2.6)		0 (0.0)		1 (5.3)		
High school	12 (31.6)		7 (36.8)		5 (26.3)		
University	25 (65.8)		12 (63.2)		13 (68.4)		
Women [n (%)]^a^	21 (55.3)		10 (52.6)		11 (57.9)		0.752^a^
Systolic blood pressure (mmHg)^†^	88–220	134.1 ± 23.6	105–220	139.7 ± 26.5	88–176	128.5 ± 19.5	0.146^†^
Diastolic blood pressure (mmHg)^†^	54–114	80.9 ± 13.9	64–108	84.7 ± 11.6	54–114	77.1 ± 15.2	0.091^†^

^†^Each value is presented as mean (standard deviation). The *p*-value for differences between groups was calculated using ^†^analysis of variance for continuous variables and ^a^χ^2^-tests for categorical variables. MMSE, Mini-Mental State Examination.

### 3.3 Cognitive function

**Figure 3:**
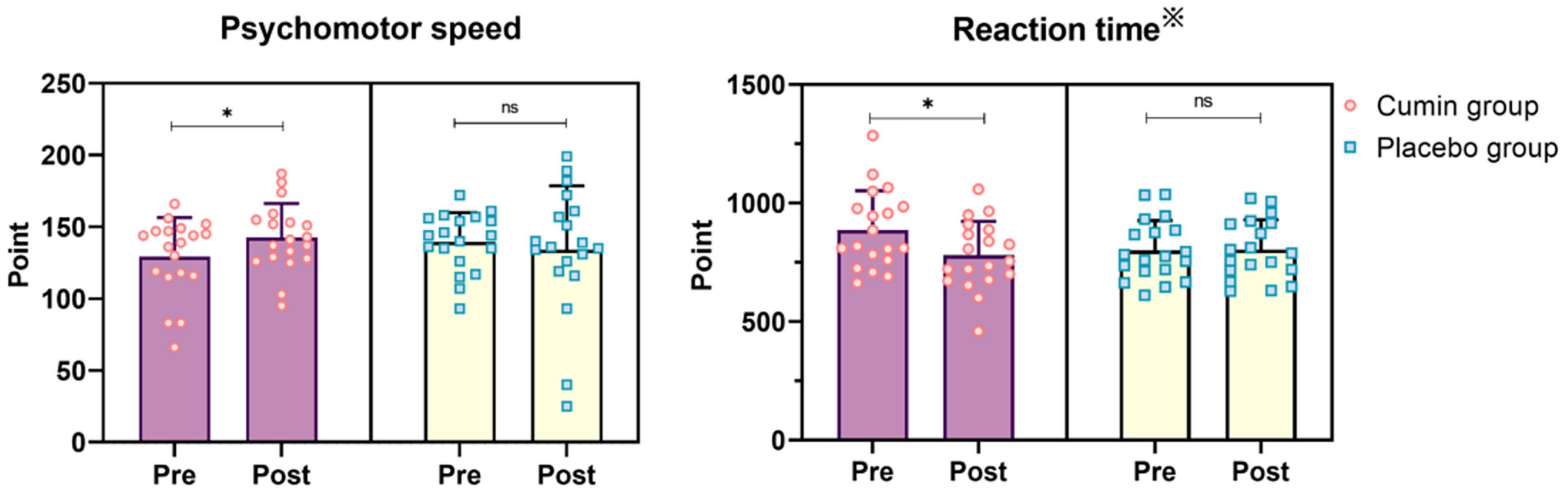
Individual tested items of psychomotor speed and reaction time. Pink circles (cumin group) and blue squares (placebo group) represent the participants’ change from baseline. *p < 0.05, ※Lower score is better.

Table 4 presents the pre- and post-intervention scores for each cognitive function, along with the results of repeated measures analysis of covariance adjusted for age and years of education. A significant group × time interaction was observed for psychomotor speed (*p* = 0.012) and reaction time (*p* = 0.022) (Table 4; Figure 3).

**Table 4:** Cognitive test by group at baseline and follow-up.

Variables	Time	CEO group (*n* = 19)				Placebo group (*n* = 19)				Interaction (groups × time)	Group effect *P*	Time effect *P*
		Mean ± SD	95% CI	Effect size (Cohen’s *d*)	Simple –Main effect *P*	Mean ± SD	95% CI	Effect size (Cohen’s *d*)	Simple –Main effect *P*			
Cognitrax, points												
Composite memory	Pre	85.4 ± 11.8	[79.7, 91.0]	0.21		87.3 ± 12.2	[81.5, 93.2]	0.21		0.973	0.858	0.091
	Post	87.9 ± 12.9	[81.7, 94.1]			89.9 ± 12.0	[84.1, 95.7]					
Verbal memory	Pre	45.8 ± 7.8	[42.0, 49.5]	0.09		45.4 ± 8.0	[41.5, 49.2]	0.41		0.275	0.962	0.086
	Post	46.5 ± 9.1	[42.1, 50.9]			48.6 ± 7.5	[45.0, 52.2]					
Visual memory	Pre	39.6 ± 4.8	[37.3, 41.9]	0.38		41.9 ± 5.1	[39.5, 44.4]	−0.12		0.126	0.639	0.430
	Post	41.4 ± 4.9	[39.0, 43.8]			41.3 ± 5.8	[38.5, 44.1]					
Psychomotor speed	Pre	129.2 ± 27.2	[116.1, 142.3]	0.43	0.048	142.6 ± 23.7	[131.2, 154.0]	−0.24	0.091	0.012		
	Post	139.5 ± 20.4	[129.6, 149.3]			133.9 ± 44.5	[112.5, 155.4]					
Reaction time※	Pre	886.2 ± 165.5	[806.4, 965.9]	0.59	0.012	778.8 ± 143.8	[709.5, 848.1]	−0.18	0.471	0.022		
	Post	799.4 ± 125.8	[738.8, 860.1]			803.3 ± 125.3	[742.9, 863.6]					
Complex attention※	Pre	26.5 ± 30.8	[11.6, 41.3]	0.32		16.5 ± 12.7	[10.3, 22.6]	−0.24		0.062	0.932	0.769
	Post	18.5 ± 16.5	[10.5, 26.4]			22.2 ± 30.9	[7.3, 37.1]					
Cognitive flexibility	Pre	15.3 ± 20.4	[5.5, 25.1]	0.07		21.8 ± 19.6	[12.3, 31.2]	0.02		0.793	0.502	0.720
	Post	16.7 ± 20.7	[6.8, 26.7]			22.2 ± 23.5	[10.9, 33.6]					
Processing speed	Pre	38.3 ± 12.0	[32.5, 44.1]	0.42		42.1 ± 12.9	[35.9, 48.3]	0.06		0.241	0.884	0.087
	Post	42.7 ± 9.1	[38.4, 47.1]			43.0 ± 15.2	[35.7, 50.3]					
Executive function	Pre	17.3 ± 19.5	[7.9, 26.7]	0.12		24.4 ± 16.3	[16.6, 32.3]	0.03		0.629	0.420	0.482
	Post	19.7 ± 20.4	[9.9, 29.5]			24.9 ± 21.0	[14.8, 35.1]					
Simple attention	Pre	29.0 ± 25.6	[16.6, 41.4]	0.41		37.0 ± 5.7	[34.3, 39.7]	−0.34		0.057	0.887	0.808
	Post	36.9 ± 9.3	[32.5, 41.4]			30.9 ± 24.8	[18.9, 42.9]					
Motor speed	Pre	90.5 ± 20.9	[80.5, 100.6]	0.29		99.6 ± 13.3	[93.2, 106.0]	−0.06		0.093	0.340	0.206
	Post	95.7 ± 14.3	[88.8, 102.6]			98.7 ± 15.6	[91.2, 106.3]					

Each value is presented as mean and standard deviation, covariate for age and educational years, *P* < 0.05, Cohen’s-*d*: |0.2 ≤ *d* < 0.5| = small, |0.5 ≤ *d* < 0.8| = moderate, |0.8 ≤ *d*| = large ※ Lower score is better.

Further analysis of simple main effects revealed that post-intervention scores for both psychomotor speed and reaction time were significantly improved compared to baseline within the CEO group (*p* = 0.048, *p* = 0.012, respectively). In contrast, no significant group × time interactions or main effects of group and time were detected for the other cognitive domains.

Additionally, effect size analysis (Cohen’s *d*) indicated that the CEO group exhibited greater improvements across multiple cognitive domains compared to the placebo group, with effect sizes as follows: visual memory (Cohen’s *d* = 0.38), psychomotor speed (Cohen’s *d* = 0.43), reaction time (Cohen’s *d* = 0.59), complex attention (Cohen’s *d* = 0.32), cognitive flexibility (Cohen’s *d* = 0.07), processing speed (Cohen’s *d* = 0.42), executive function (Cohen’s *d* = 0.12), simple attention (Cohen’s *d* = 0.41) and motor speed (Cohen’s *d* = 0.29).

### 3.4 Blood biomarker analysis

Table 5 present the pre- and post-intervention biomarker measurements, along with the results of repeated measures analysis of varience. In this study, plasma TNF-*α*, IL-6, Aβ_1-40_, Aβ_1-42_ and BDNF were measured to analyze the effect of CEO supplementation on biomarkers related to cognitive function and aging. Before *post-hoc* analysis on, data cleaning for each secondary outcomes excluded one record of IL-6 in the CEO group.

**Table 5:** Blood test by group at baseline and follow-up.

Variables	Unit	Time	CEO group (*n* = 19)				Placebo group (*n* = 19)				Interaction (groups × time)	Group effect *P*	Time effect *P*
			*n*	Mean ± SD	95% CI	Effect size (Cohen ‘s *d*)	*n*	Mean ± SD	95% CI	Effect size (cohen ‘s *d*)			
d-Roms ※	U. CARR	Pre	19	413.9 ± 87.7	[371.6, 456.2]	0.43	19	386.5 ± 58.9	[358.1, 414.9]	0.41	0.455	0.398	0.001
		Post		374.4 ± 96.6	[327.8, 420.9]			360.9 ± 66.4	[328.9, 393.0]				
BAP	μmol/L	Pre	19	2655.8 ± 164.0	[2576.8, 2734.9]	0.66	19	2599.6 ± 175.9	[2514.8, 2684.4]	1.38	0.060	0.923	<0.001
		Post		2775.1 ± 194.3	[2681.4, 2868.8]			2840.8 ± 173.4	[2757.2, 2924.4]				
Aβ_1-40_ /Aβ_1-42_	Ratio	Pre	19	14.4 ± 4.0	[8.6, 13.0]	−0.85	19	15.7 ± 3.1	[10.6, 12.9]	−1.41	0.802	0.290	<0.001
		Post		10.8 ± 4.5	[12.5, 16.4]			11.8 ± 2.4	[14.2, 17.2]				
BDNF	pg/mL	Pre	19	528.4 ± 255.9	[405.1, 651.8]	1.42	19	481.5 ± 327.0	[323.9, 639.1]	1.69	0.218	0.870	<0.001
		Post		970.5 ± 360.2	[796.9, 1144.1]			1048.0 ± 344.7	[881.9, 1214.1]				
IL-6 ※	pg/mL	Pre	18	28.0 ± 13.4	[21.4, 34.7]	0.93	19	26.0 ± 6.2	[23.0, 29.0]	0.25	0.071	0.500	0.003
		Post		16.1 ± 12.2	[10.0, 22.2]			22.8 ± 16.9	[14.7, 31.0]				
TNF-α ※	pg/mL	Pre	19	32.6 ± 26.1	[20.0, 45.2]	0.49	19	34.3 ± 25.9	[21.8, 46.7]	0.63	0.740	0.923	<0.001
		Post		21.7 ± 17.2	[13.4, 30.0]			21.3 ± 13.8	[14.6, 27.9]				

Each value is presented as mean and standard deviation, covariate for age and educational years, *P* < 0.05, Cohen’s-*d*: |0.2 ≤ *d* < 0.5| = small, |0.5 ≤ *d* < 0.8| = moderate, |0.8 ≤ *d*| = large ※ Lower score is better.

A significant main effect of time was observed for all blood biomarkers (*p* < 0.05). However, there were no significant group × time interactions. The *p* values for each outcome were as follows: d-Roms (*p* = 0.455), BAP (*p* = 0.060), Aβ_1–40_/Aβ_1-42_ (*p* = 0.802), BDNF (*p* = 0.218), IL-6 (*p* = 0.071), TNF-α (*p* = 0.740). The CEO group showed smaller effect sizes for BAP (Cohen’s *d* = 0.66), Aβ_1–40_/Aβ_1–42_ (Cohen’s *d* = 0.85), BDNF (Cohen’s *d* = 1.42), and TNF-α (Cohen’s *d* = 0.49) compared to the placebo group. However, the effect sizes for d-ROMs (Cohen’s *d* = 0.43) and IL-6 (Cohen’s *d* = 0.93) were larger than in the placebo group.

### 3.5 Quality of life

The pre- and post-intervention points of each tests for quality of life were showed at Supplementary Table 1. A significant group × time interaction was observed for Role physical (*p* = 0.048), besides, simple main effects revealed that the score were significantly declined compared to baseline within the placebo group (*p* = 0.031). No other significant group × time interactions were detected.

### 3.6 CEO and its main active component, cuminaldehyde, induced the inhibition of Aβ_42_-induced cell death on human neuroblastoma SH-SY5Y cells

To evaluate the neuroprotective effect of CEO and cuminaldehyde, SH-SY5Y cells were pre-treated with CEO (25 and 50 μg/mL) or cuminaldehyde (3.705 and 7.41 μg/mL) for 24 h. And then, Aβ42 was added (final concentration: 2.5 μM) and co-treated with CEO or cuminaldehyde for 24 h; and cell viability was measured with the MTT assay. As shown in figure Supplementary Figure 1, the Aβ42-treated group showed a significantly reduction in cell viability compared to the non-treated group (56.8 ± 1.1%). In contrast, pre-treatment with CEO (25 and 50 μg/mL) and cuminaldehyde (3.705 and 7.41 μg/mL) ameliorated Aβ42-induced cytotoxicity (62.4 ± 1.4% and 67.6 ± 1.8%, respectively, *p* < 0.01) (Supplementary Figures 1A,B). Moreover, our results also showed that the cuminaldehyde, the main active component of CEO, treatment (3.705 and 7.41 μg/mL) ameliorated Aβ42-induced cytotoxicity (68.2 ± 1.2% and 75.9 ± 2.3%, respectively, *p* < 0.01) (Supplementary Figure 1B).

## 4 Discussion

Cognitive decline threatens independence and quality of life during old age (27, 28). Cross-sectional studies have shown that cognitive function starts declining gradually in the late 20s, and the decline is accelerated by neurodegenerative diseases, including Alzheimer’s disease (29). Therefore, early intervention strategies aimed at preserving cognitive function are critical for promoting healthy aging and mitigating age-related cognitive impairment (30).

This study is among the first to examine the effects of CEO, which is rich in cuminaldehyde, on cognitive function in older adults. Participants who received a daily dose of 25 mg of cuminaldehyde for 12 weeks demonstrated significant differences (interaction: group × time) in psychomotor speed and reaction time compared to the placebo group (*p* = 0.012, *p* = 0.022) (Table 4; Figure 3). Moreover, a simple main effects analysis confirmed that these improvements were statistically significant in the CEO group (*p* = 0.048, *p* = 0.012), whereas no significant changes were observed in the placebo group (*p* = 0.091, *p* = 0.471). Effect size analysis further supported these results, indicating a greater effect in the CEO group (Cohen’s *d* = 0.43 for psychomotor speed and Cohen’s *d* = 0.59 for reaction time), compared to the placebo group (Cohen’s *d* = −0.24, Cohen’s *d* = −0.18) (Table 4).

The psychomotor speed domain in Cognitrax is determined by performance in the Finger Tapping Test and the Symbol Digit Coding test, both of which assess an individual’s ability to perceive, attend to, and respond to complex visual-perceptual information while performing motor speed and fine motor coordination tasks. The domain score for reaction time, defined as “how quickly the subject can react, in milliseconds, to a simple and increasingly complex direction set” (23). Psychomotor slowing is a hallmark of normal aging and a core feature of dementia (31). Previous studies have shown that declines in psychomotor processing can influence other cognitive domains and contribute to broader cognitive impairments, including deficits in verbal fluency (30–32). Additionally, psychomotor speed is integral to various daily activities, such as reducing fall risk, driving, playing musical instruments, and executing other fine motor tasks (33). Given its importance in daily life, maintaining psychomotor speed is critical for older adults. The observed improvements in psychomotor and motor speed suggest that CEO supplementation may enhance neurocognitive processing efficiency and fine motor function in older adults, highlighting the potential of CEO as a natural intervention for supporting cognitive health and functional independence in aging populations.

In this study, the complex attention domain scores changed from 26.5 to 18.5 (Cohen’s *d* = 0.32) in the CEO group and from 16.5 to 22.2 (Cohen’s *d* = −0.24) in the placebo group, with lower scores indicating better performance. Although these results were not statistically significant, they align with previous findings, suggesting potential cognitive benefits of CEO. Changes in attention can influence various cognitive functions, including multitasking, working memory, problem-solving, and visual processing (34). In the placebo group, only one item showed a positive effect size of small or greater, while three items displayed negative effect sizes. In contrast, the CEO group did not exhibit any negative effect sizes, with eight items showing small or larger positive effect sizes (Table 4). Despite the absence of a significant interaction effect, the CEO group demonstrated an improvement in overall cognitive function.

As shown in Table 5, exploratory analyses of blood biomarkers revealed no significant group × time interactions for any biomarker (all *p* > 0.05). Significant main effects of time were observed for all biomarkers included in the present study (IL-6, *p* = 0.003; all others, *p* < 0.001), indicating changes over the course of the intervention in both groups. Supplementary Table 2 shows the coefficient of variation for each biomarker in the blood biomarker analysis. Other than IL-6 (Pre: 0.107, Post: 0.376), relatively small inter-assay coefficient suggest high data accuracy. While, high intra-assay coefficient of variation was considered to attribute to inter-individual variability. Within-group effect sizes are provided in Table 5; the magnitude and direction of changes were not consistently greater in the CEO group than in the placebo group. Chronic inflammation, typified by elevated levels of IL-6 and TNF-*α*, has been associated with age-related diseases, including cellular senescence, immune dysfunction, and cognitive decline (2). Increased IL-6 levels have been linked to cognitive decline in healthy elderly individuals (35). Chi et al. (36) found that chronic inflammation was related to cognitive decline in domains such as memory and psychomotor speed. Furthermore, Palta et al. (37) observed that higher serum IL-6 levels were associated with a domain-specific decline in psychomotor speed, potentially leading to changes in brain microvascular function, which impairs reaction time and mental processing speed. The observed reduction in IL-6 suggests that CEO supplementation for 12 weeks may alleviate chronic inflammation associated with aging, thereby mitigating cognitive decline, particularly in domains such as psychomotor speed and reaction time. Possible explanation for this might be that increasing IL-6 can also be caused by senile plaques formed around microglia and strocytes in pathological or aged brain (38, 39). Further evidence linking biomarkers to cognitive function supports the association between inflammation and specific cognitive domains, particularly psychomotor speed and reaction time. Because no biomarker demonstrated a statistically significant group × time interaction, the present data do not provide confirmatory evidence that CEO uniquely modulates these biomarkers. The numerically larger decrease in IL-6 in the CEO group (p for interaction = 0.071) may warrant further investigation; however, this finding should be considered exploratory and hypothesis-generating, particularly given the limited sample size and the assessment of multiple biomarkers.

To further understand the impact of CEO on cognitive functions, we conducted a component analysis. Based on component analysis, we can discuss how CEO and its components may enhance neuroprotection or modulate the production of factors associated with neurological dysfunction, such as neurotransmitters and neuroinflammation. Cuminaldehyde, a member of the class of benzaldehydes, was a main aromatic component of CEO consistent with previous report (40). Therefore, cuminaldehyde was expected to be responsible for remarkable bioactivity of CEO. In terms of its neuroprotective effect, an *in vivo* study conducted on aged mice demonstrated that cuminaldehyde improved learning, memory, and locomotor activity. Furthermore, after 30 days of cuminaldehyde consumption modulated IL-6, TNF-*α*, catecholamines, and gene expression of *Bdnf*, *Icam*, *ApoE*, and *Il-6* in the brain was observed (12). Our results showed partial consistency with this in vivo study. *p-*Cymene, *γ*-terpinene, and *β*-pinene are monoterpenes commonly found in essential oils from various plant-based foods such as vegetables, fruits, and spices. Several in vivo studies have highlighted the pharmacological potential of these monoterpenes. *γ*-terpinene has been studied for its effects on acute inflammation, oxidation, and apoptosis in brain tissue in mice (41, 42). However, there are no reports on their chronic efficacy for cognitive function. *β*-pinene has shown antidepressant-like activity through the adrenergic β-receptor and dopaminergic D1 receptor (43). Additionally, an intervention with 50–200 mg/kg of β-pinene for 21 days in Alzheimer’s disease model rats alleviated mitochondrial dysfunction and oxidative stress in the brain, leading to improvements in cognitive function (44). However, it is unlikely that β-pinene alone accounts for the observed effects in our study, as the administered doses in these studies (50–200 mg/kg) were considerably higher than the equivalent dose of 3.8 mg/60 kg/day in our study. Consequently, it can be inferred that cuminaldehyde is the dominant active component in CEO, while other minor components, such as *p-*cymene, β-pinene, and γ-terpinene, may contribute to its efficacy as natural complex. To verify this suggestion, it was necessary to confirm that the protective effect of CEO on cell viability against Aβ-induced neurodegeneration, a typical and widely used *in vitro* system for evaluating broad neurogenerative processes, (Supplementary Figure 1A) encompassed that of cuminaldehyde at the corresponding concentration (Supplementary Figure 1B). In conclusion, CEO was probably identified as the dominant active component responsible for the cognitive function in the elderly.

A practical implication of the findings in this study is the potential benefit of incorporating cuminaldehyde from cumin seeds into a healthy diet to prevent cognitive decline. The 12-week consumption of the test food, containing 25 mg of cuminaldehyde, demonstrated a protective effect on cognitive decline. The essential oil derived from cumin seeds in this study contained approximately 40% (w/v) cuminaldehyde, along with other volatile components. Typically, 2.3–5.7% of essential oil is distilled from cumin seeds (45). This suggests that incorporating about 1.1–2.7 g of cumin seed ingredients into a daily diet could help prevent cognitive decline. A dietary survey in India found that 60.2% of urban residents and 30.3% of rural residents used 1–3 g of cumin seed powder in their daily diet (46). This aligns with anwobservational study in Singapore, which suggested an association between the consumption of curcumin-rich curry and improved MMSE scores (20). It is important to note that a dietary intake of cumin seeds corresponding to 25 mg of cuminaldehyde, especially when included in spice-infused dishes such as curry, and supplementation with CEO, could be a feasible approach, to help prevent cognitive decline in healthy individuals.

Several limitations should be acknowledged. First, the final analyzed sample size (*n* = 38; 19 per group) was smaller than intended because of dropout and missing data, which may have reduced statistical power and increased the risk of type II error. Therefore, non-significant findings—particularly in cognitive domains other than psychomotor speed and reaction time—should be interpreted cautiously and should not be considered definitive evidence of no intervention effect. Future studies with larger, adequately powered samples are warranted. Secondly, the dietary intake of spices could have been a confounding factor. The participants were instructed and provided with a semi-quantitative paper-based questionnaire comprising both fixed-response items and free-form response items as follows: “Do not consume these meals for as long as possible. In the event of consumption, write down the items and verify the amount accordingly. The items include curry rice, curry udon, curry pilaf, curry pan, and others.” Over the course of 12 weeks, no check was reported (data not shown). However, given the self-reported nature of the questionnaire, the possibility of diet as an unmeasured confounding variable and the potential for reporting inaccuracies, as well as measurement error, cannot be ruled out. Thirdly, the *in vitro* findings cannot be directly extrapolated to clinical Alzheimer’s pathology. Best to our knowledge, no pharmacokinetic studies have been reported on cuminaldehyde in both mouse and human. In addition, the 12 weeks trial duration may be insufficient to detect meaningful changes in Aβ ratios and BDNF. Importantly, our trial enrolled cognitively healthy older adults with high baseline MMSE scores; therefore, the present findings should not be interpreted as evidence of neuroprotection against Alzheimer’s-like pathology or enhanced Aβ clearance in humans. In addition, our exploratory blood biomarker analyses did not show significant group × time interactions, and peripheral biomarkers may not directly reflect central nervous system pathology. Accordingly, the observed cognitive changes may be attributable to broader physiological effects of CEO (e.g., systemic anti-inflammatory and anti-oxidative actions) that could indirectly support cognitive performance. Future trials in individuals with mild cognitive impairment or biomarker-confirmed pathology, together with mechanistic endpoints, are needed to determine whether CEO modulates Alzheimer’s-related pathways in humans. Finally, multiple biomarker outcomes were tested without formal multiplicity correction, there is an increased possibility of type I error. Accordingly, nominally significant findings should be considered exploratory and require confirmation in adequately powered trials.

Conclusionly, this study evaluated the efficacy of short term daily consumption of CEO, primarily composed of cuminaldehyde, on cognitive function in the elderly. The results indicated that CEO can enhance several aspects of cognitive function in healthy elderly Japanese individuals after a 12-week intervention. Specifically, significant improvements were observed in the domains of psychomotor speed and reaction time when compared to the placebo group.

## Data Availability

The raw data supporting the conclusions of this article will be made available by the authors, without undue reservation.

## Supplementary material

The Supplementary materials for this article can be found online at: https://www.frontiersin.org/articles/10.3389/fnut.2026.1784027/full#supplementary-material
