# Supplementary material for: Daily intake of cuminaldehyde-rich cumin essential oil improves cognitive function in healthy elderly Japanese adults: a randomized, double-blind, placebo-controlled pilot study
Source: Front Nutr. 2026 Apr 20;13:1784027. doi: 10.3389/fnut.2026.1784027 (PMC13135935; doi:10.3389/fnut.2026.1784027)
Supplement: Supplementary file 1 [file Table_1.docx]

**
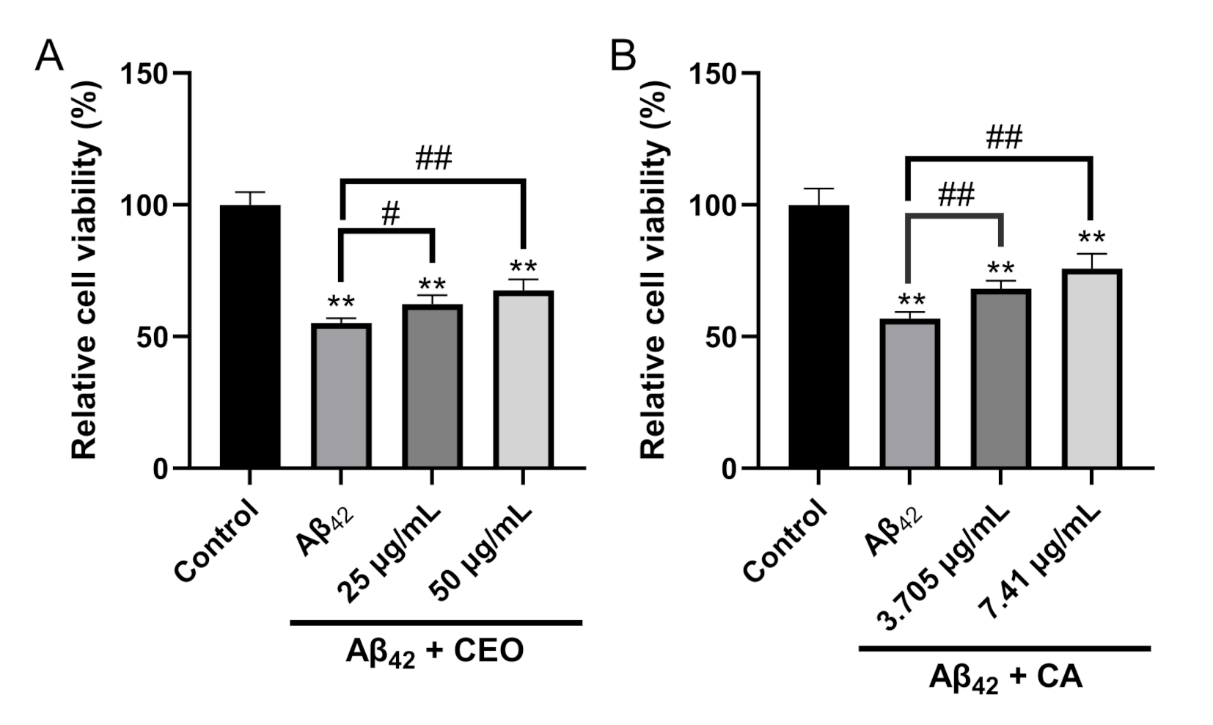
**

**Supplementary figure 1**. **Effect of (A) CEO and (B) cuminaldehyde (CA) on Amyloid-β(Aβ)_42_-induced changes in human neuroblastoma SH-SY5Y cells.**

SH-SY5Y cells were pretreated with CEO or CA for 24 hr and then treated with 2.5 μM Aβ_42_ for 24 hr to evaluate neuroprotection. Statistical significance was assessed using one-way ANOVA followed by Tukey’s post-hoc test. Each bar represents the mean ± SEM (n = 5 independent experiments). ** *p* < 0.01 vs. control cells, ## *p* < 0.01 vs. Aβ_42_-treated cells.

**Supplementary Table 1.** **Secondary outcomes by group at baseline and follow-up**

Note: Each value is presented as mean and standard deviation, covariate for age and educational years, P < 0.05, ※ Lower score is better or positive mood.

**Supplementary Table 2. The coefficient of variation for each biomarker in the blood biomarker analysis.**

The inter-assay coefficient of variation for biomarkers other than IL-6 (Pre: 0.107, Post: 0.376) was relatively small (Aβ_40_: 0.150 (Pre), 0.063 (Post); Aβ_42_: 0.167 (Pre), 0.068 (Post); BDNF: 0.179 (Pre), 0.093 (Post); TNF-α: 0.139 (Pre), 0.103 (Post)). On the other hand, the intra-assay coefficient of variation in our blood biomarker analysis was relatively high (Aβ_40_: 0.234 (Pre), 0.192 (Post); Aβ_42_: 0.603 (Pre), 0.234 (Post); BDNF: 0.562 (Pre), 0.342 (Post); TNF-α: 0.768 (Pre), 0.706 (Post); IL-6: 0.371 (Pre), 2.189 (Post)).

**Supplementary Table 3. Results of non-parametric between-group comparisons of Cognitrax scores**

Note: Statistical significance between groups was assessed using Mann-Whitney U test, P < 0.05

**Supplementary Table 4. Results of non-parametric between-group comparisons of biomarkers**

Note: Statistical significance between groups was assessed using Mann-Whitney U test, P < 0.05
